# Supplementation of complex natural feed additive containing (*C. militaris*, probiotics and red ginseng by-product) on rumen-fermentation, growth performance and carcass characteristics in Korean native steers

**DOI:** 10.3389/fvets.2023.1300518

**Published:** 2024-01-15

**Authors:** Mun-Su Ju, Yong-Ho Jo, Yoo-Rae Kim, Jalil Ghassemi Nejad, Jang-Gu Lee, Hong-Gu Lee

**Affiliations:** ^1^Laboratory of Animal Nutrition, Physiology and Proteomics, Department of Animal Science and Technology, Konkuk University, Seoul, Republic of Korea; ^2^DM Bio Co., Ltd., Jellonam-do, Republic of Korea

**Keywords:** *C. militaris*, probiotics, rumen, Korean-native steers, feed additive

## Abstract

This study evaluated the effects of a complex natural feed additive on rumen fermentation, carcass characteristics and growth performance in Korean-native steers. In this study, *in vitro* and *in vivo* experiment were conducted. Seven different levels of complex natural feed additive (CA) were added to the buffered rumen fluid using Ankom^RF^ gas production system for 12, 24 and 48 h. All experimental data were analyzed by mixed procedure of SAS. Total gas production increased in the CA groups, with the highest response observed in the 0.06% group at 48 h of incubation (linear, *p* = 0.02; quadratic, *p* < 0.01). Regarding rumen fermentation parameters, the total volatile fatty acid (TVFA) tended to increase in all the CA groups (*p* = 0.07). The concentrations of butyrate, iso-butyrate, and iso-valerate significantly increased in all treatment groups (*p* < 0.05). In the *in vivo* experiment, 23 Korean-native steers were allocated to two groups: (1) Control and (2) Treatment; control +0.07% CA (DM basis), in a randomized complete-block design and blocked by body weight (ave. body weight = 641.96 kg ± 62.51 kg, *p* = 0.80) and feed intake (ave. feed intake = 13.96 kg ± 0.74 kg, *p* = 0.08) lasted for 252 days. Average daily gain decreased in the treatment group (*p* < 0.01). Backfat thickness significantly decreased in the CA group (*p* = 0.03), whereas meat color tended to increase (*p* = 0.07). In conclusion, in the *in vitro* experiment, the inclusion of complex natural feed additive decreased methane proportion and tended to increase TVFA production, but supplementation to Korean native steers decreased average daily gain and backfat thickness.

## 1 Introduction

With the increase in the global population, growth of the global economy, and urbanization, there has been an increased demand for livestock products, including meat, milk, and eggs. These phenomena put pressure on the livestock industry to meet global expectations (1). Recently, animal producers have shown interest in natural compounds to improve animal physiological state and health (2). There are numerous natural compounds that can serve as alternatives to growth-promoting antibiotics to enhance animal health and well-being (3). One of these alternatives is probiotics, referred to as ‘direct-fed microbial’ (DFM). DFM refers to living microorganisms utilized to enhance livestock performance by modifying the microbial composition within the animal intestinal tract (4). Probiotics are employed in the ruminant industry to modulate the microflora and fermentation activities for the benefit of the host (5). Lactic acid-producing bacteria, lactic acid-utilizing bacteria, yeast, and fungi are commonly utilized as probiotics in ruminants (5). DFMs act in both the intestinal tract and post-gastrointestinal region. Several studies have reported that these microorganisms not only enhance performance but also modulate the immune response of animals (6–8).

*Cordyceps militaris* (*C. militaris*) is entomopathogenic fungus that has been used for the last 300 years in East Asia due to its adaptogenic and tonic effects (9). *Cordyceps* spp. contain several bioactive compounds such as cordycepic acid (D-mannitol), cordycepin, ergosterol and adenosine (10, 11). *C. militaris* has been shown to improve *in-vitro* rumen fermentation, leading to an increase in volatile fatty acids (VFA), cellulose digestion, and cellulolytic enzyme activities (12, 13). *Panax ginseng* belongs to the Araliaceae family, which is an herbaceous plant and one of the most precious plant products in Northeast Asia (14). Red ginseng is produced by steaming fresh ginseng, known to contain pharmaceutical efficacy due to its major bioactive constituent (triterpene saponin), also called ginsenosides (15). This manufacturing process is known to enhance the saponin content in ginseng (16). Due to the medicinal efficacy of red ginseng, there has been an increase in the production of red ginseng, leading to the production of about 8,000 tons of by-products (17). Regardless of extracting its bioactive compounds using solvents like water or, alcohol, are known to contain bioactive compounds such as ginsenosides (15). By-products of red ginseng continue to be produced as the market is increasing, and several studies have indicated that these could be used as additives in the animal feed industry (17, 18).

Recently, phytogenic feed additives have been recognized for their ability to influence feed quality, animal health, as well as animal products. Several studies have shown that the individual components of natural feed additives can have various positive effects on animals (19–21). It is expected that a combination of these components will work synergistically to enhance outcomes when included in animal diets. To our knowledge, no previous work has been conducted to evaluate the performances of cattle using a mixture of these compounds. Therefore, this study aimed to evaluate the effects of combination of probiotics and natural feed additives on the physiological aspects and growth performance of Korean-native cattle.

## 2 Materials and methods

### 2.1 Preparing complex natural feed additive (CA)

The complex natural feed additive, containing (probiotics, red ginseng by-product and *C. militaris*) was obtained from DM bio-Co., LTd (Jeollanam-do, Korea). Probiotics were inoculated into a sterilized medium composed of red ginseng by-product, soy-bean meal, distillers’ grain, wheat bran and condensed molasses soluble (CMS). The probiotics used in this additive included *Bacillus subtilis* (1.0 × 10^11^ CFU/g), *Aspergillus oryzae* (1.0 × 10^10^ CFU/g), *Saccharomyces cerevisiae* (1.0 × 10^11^ CFU/g), *Lactobacillus acidophillus* (1.0 × 10^10^ CFU/g), and *Streptococcus thermophilus* (1.0 × 10^11^ CFU/g). After inoculating the probiotics, the mixture was dried at low temperature and *C. militaris* (20%) was added to the total volume of the complex natural feed additive (Korea. Patent No. 10–1137893).

### 2.2 Chemical analysis

Experimental diets were finely milled through a 250 ~ 500 μm screen and analyzed for chemical composition (Table 1). All samples were dried in an air-drying oven at 60°C for 48 h to determine the chemical composition. The chemical analysis for ether extract (EE; Method 920.39), crude protein (CP; Method 954.01), and crude ash (Ash; Method 942.05) of diets were followed methods of Association of Official Analytical Chemists (AOAC, 1990). The aNDF and ADF were analyzed using the Van Soest method (22).

**Table 1:** Chemical composition of experimental diet.

Items^1^	Basal diet^2^	Complex natural feed additive
Ingredients (%)		
Basal concentration	24.03	-
Soybean meal	3.03	15.00
Corn flake	30.41	21.00
DDGS	-	9.00
Wheat bran	-	15.00
*Cordyceps militaris*	-	20.00
Red ginseng by-product	-	20.00
Rice straw	12.00	-
Water	29.91	-
Vitamin and mineral mix	0.01	-
Probiotics, (cfu/g)	0.14	-
*Saccharomyces cerevisiae*	-	1.0 × 10^11^
*Bacillus subtilis*	-	1.0 × 10^11^
*Lactobacillus acidophilus*	-	1.0 × 10^10^
*Streptococcus thermophilus*	-	1.0 × 10^11^
Limestone	0.20	-
Salt	0.27	-
Total	100.00	100.00
Nutrient composition (% of DM)		
DM (%)	77.26	44.06
CP	14.07	21.87
EE	4.56	2.77
NDF	51.75	36.74
ADF	23.02	12.28
NDICP	10.71	18.84
ADICP	6.67	19.62
Macro minerals (% of DM)		
Ca	0.81	
P	0.47	

^1^ DDGS, Dried distiller’s grain with solubles; CP, Crude Protein; EE, Ether Extract; NDF, Neutral detergent fiber; ADF, Acid detergent fiber; NDICP, Neutral detergent insoluble protein; ADICP, Acid detergent insoluble protein; Ca, Calcium; P, Phosphorus. ^2^ Basal diet, Diet was provided as a total mixed fermented ration.

### 2.3 *In vitro* experiment

The basal diets used in the *in-vivo* study were milled through a 1 mm screen and used as a substrate in this experiment. Before collecting rumen fluid, the experimental diet was given to cannulated cows for adaptation for 7 days. After adaptation period, rumen fluid was obtained from 2 cannulated Holstein cows, and an equal amount of rumen fluid was obtained from each animal. It was then filtered through a 250 μm pore-sized nylon filter (Shanghai Bolting Cloth Manufacturing Co., Ltd., Shanghai, China). The filtered fluid was mixed with Menke’s buffer in a 1:3 ratio (23). For the *in-vitro* experiment, we used a wireless gas pressure monitoring system, the Ankom^RF^ gas production system (ANKOM Technology, Macedon, NY, USA) (24). Each bottle was filled with 125 mL of buffered solution and added ANKOM bags (R510; Ankom Technology, USA). A substrate (1.25 g) was placed in each nylon bag and mixed with CA at levels of 0, 0.015, 0.03, 0.06, 0.12, 0.24, and 0.48% of dry matter basis. Maintaining an anaerobic state in buffered rumen fluid, ultra-purity argon gas (99.999%) was used in this experiment (25). Incubation was carried out in a shaking incubator (JSSI-300C, JSR, Gongju, Korea) at 39°C for 48 h at 85 rpm. A total of 3 experiments were conducted, and each experiment consisted of duplicated treatment bottles and two blank bottles, totaling 54. The blank bottles were used to correct data for Total gas production (TGP), *In-vitro*, dry matter digestibility (IVDMD), and total volatile fatty acids (TVFA).

The cumulative pressure was set to be recorded every 20 min using the official manufacturer’s software (Gas Pressure Monitor, Ankom Technology, Macedon, NY, USA) until the end of the incubation. The kinetics of gas production were analyzed using a single-pool and single-lag exponential model (26).


$$V_{t}=0\;\left(0\leq T\leq L\right).$$

$$V_{t}=V_{\max}*(1-\exp\left(-K*\left(T-L\right))\right)\;\left(T\geq L\right).$$

Where V_t_ is the gas produced at a specific time T (mL), V_max_ is the asymptotic gas production (h^−1^), Exp is the exponential function, K is the fractional rate of gas production (h^−1^), L is the discrete lag time (h), and t is the time after the initiation of incubation (h). For CH_4_ analysis, 3 mL of gas was sampled at 12, 24, and 48 h into a vacuum container (Labco Ltd., High Wycombe, UK). CH_4_ analysis was conducted using a gas chromatography system (HP 6890 series GC system; Agilent Technologies Inc., Santa Clara, CA, USA). A thermal conductivity detector and capillary column (HP-PLOT/Q; Agilent Technologies Inc., Santa Clara, CA, USA) were used in the gas chromatography system, and a standard gas mixture (H_2_ 1.0%, CO_2_ 20.1%, CH_4_ 10.1%, and N_2_ 19.9% in He) was used to quantify CH_4_ concentration. After the experiment completed, the nylon bags were removed to measure IVDMD by calculating the weight loss of the substrate. Ruminal pH was measured using a pH meter (S20 SevenEasy pH, Mettler Toledo, Greifensee, Switzerland). For the measurement of volatile fatty acid (VFA) and NH_3_-N, rumen fluids were collected into 50 mL tube and then centrifuged at 2,000 g at 4°C for 10 min to deposit feed particles. The 5 mL of the supernatant rumen fluid were collected in 15 mL tubes and mixed with 1 mL of a 2% HgCl_2_ (wt/vol) and 25% meta-phosphoric acid (wt/vol) solution for NH_3_-N and VFA analysis. For VFA analysis, 1.4 mL of supernatant was collected to 2 mL tube and centrifuged at 20,000 g at 4°C for 20 min. After that a volume of 25 μL of 1% (wt/vol) pivalic acid was added to sample for using an internal standard and stored at brown vial until analysis. Profiles of VFA were analyzed using the gas chromatography (HP 6890 series GC system; Agilent Technologies Inc., Santa Clara, CA, USA) equipped with a flame ionization detector and a capillary column (DB-FFAP; Agilent Technologies Inc., Santa Clara, CA, USA).

For NH_3_-N analysis, previously centrifuged 600 μL samples was transferred to a 1.5 mL tubes and centrifuged again at 20,000 g at 4°C for 20 min. Then, 500 μL of supernatant were collected into new 1.5 mL tubes. NH_3_-N concentration was measured using the catalyzed indophenol reaction (27) using spectrophotometry (Synergy2; Biotek Instruments, Inc., Winooski, VT, USA).

### 2.4 Animals and experimental condition (*In-vivo*, experiment)

All the animal experimental procedure were approved by the Institute of Animal Care and Use Committee of Konkuk University (approval no. KU22079). 23-Korean native steers (Initial body weight 641.96 ± 62.51 kg; age 24.22 ± 0.93 months) were used for the experiment. Before starting the experiment, a total of 5 days, feed intake was checked daily for each pen by weighing orts to measure dry matter intake (DMI). Initial Body Weight (IBW) was measured using a digital scale (CAS Co. Ltd., Seoul, Korea). The animals were allocated to 2 dietary treatments, control (without CA) and treatment (0.07% of CA supplementation on a dry matter basis), using a randomized complete block design considering factors such as feed intake, age, and IBW. After a 7 days adaptation period, the total experiment lasted for 252 days, during which animals were fed twice daily (07,00 and 16,00). Mineral blocks and water were provided *ad libitum*. The offered diets and refusals were measured for three consecutive days each month to calculate the pen’s DMI. CA was provided to the experimental animals by top-dressing onto basal diet at every 16:00 feeding time.

### 2.5 Blood sampling and metabolic profiles analysis

Blood samples were taken from each cow through the jugular vein on day 144. For complete blood cell count test, blood was collected into EDTA-containing tubes (Becton Dickinson, Franklin Lakes, NJ, USA) and analyzed using the HM2 analyzer (Abaxis, Union City, CA, USA). For serum analysis, blood was collected into heparin-containing tubes (Becton Dickinson, Franklin Lakes, NJ, USA) and centrifuged at 3,000 rpm for 15 min to extract serum. Serum samples were stored at −80°C until analysis. Fuji dry-chem slides (DRI-CHEM 7000i, Fuji Film, Tokyo, Japan) was used to determine serum metabolite concentration.

### 2.6 Carcass traits

Slaughtering procedure of experimental animals were conducted at a commercial slaughterhouse in Chungcheongnam-do, Korea. In each group, the slaughter of four animals was carried out sequentially on days 223, 244, and 255, respectively. After slaughter and a 24-h chilling process, the evaluation was conducted, including yield grade (carcass weight, back fat thickness, and ribeye area) and carcass quality grade (marbling score, meat color, texture, and maturity). Carcass traits scores were assessed according to the Korean Carcass Grading System of the Korea Institute of Animal Products Quality Evaluation (KAPE, 2019). The quality grade was assessed on the surface of the longissimus thoracis muscles in the 13th rib section.

### 2.7 Statistical analysis

All the data from the experiment were statistically analyzed using SAS University Studio (SAS Institute, Inc., Cary, NC, USA). The rumen fermentation data were analyzed using the PROC MIXED procedure of SAS, and the statistical model was as follows:


$$Y_{ij}=\mu +T_{i}+R_{j}+e_{ij}.$$

**Figure 1:**
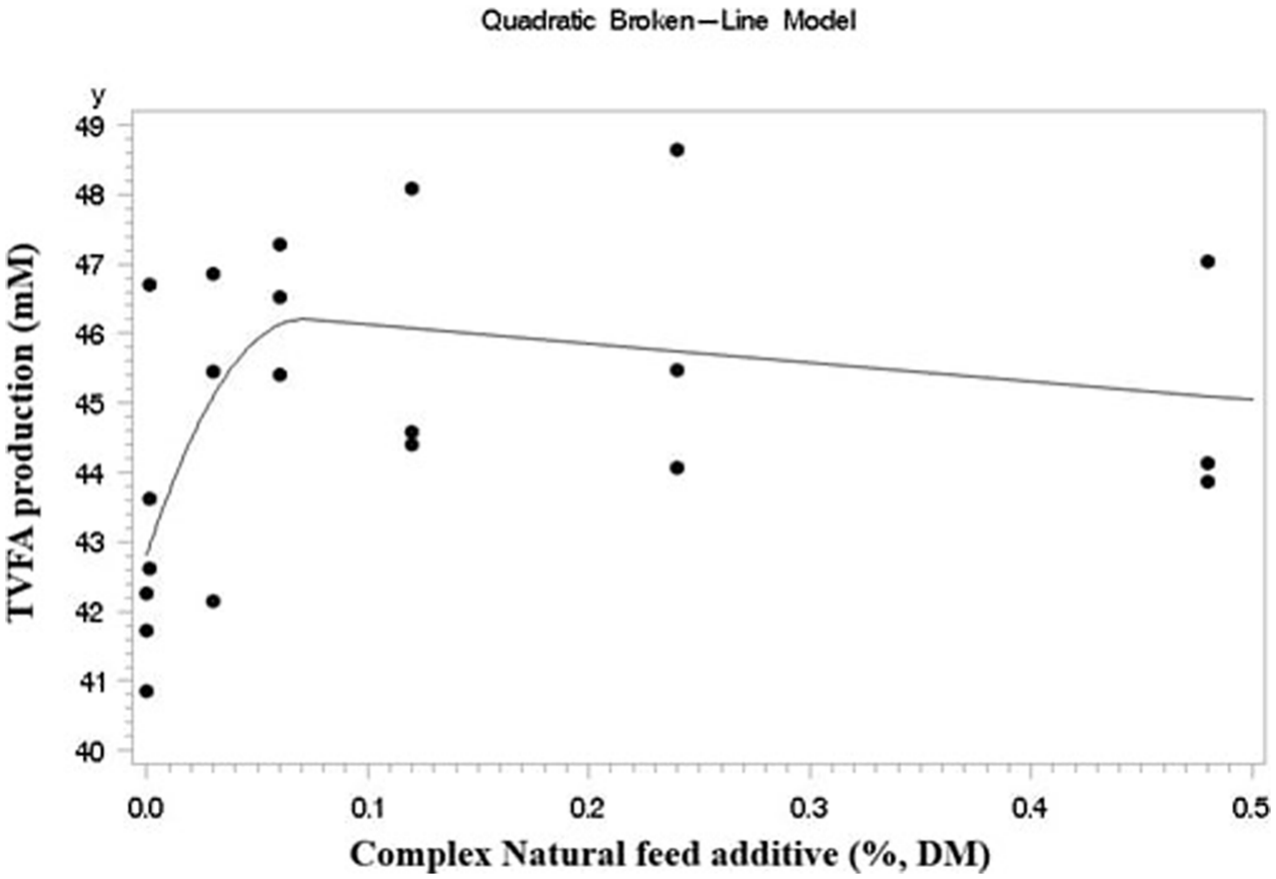
Broken-line regression models fitted to data of TVFA production expressed as (y), in response to CA inclusion level %DM as (x) *Y* = 46.2021–684.7 (x - 0.073)^2^ (x > 0.073), *Y* = 46.2021–2.6937x (x < 0.073) (*r*^2^ = 0.37; *p* = 0.045).

Where Y_ij_ is the observation from the experimental unit, μ is the overall mean, T_i_ is the fixed effect of the dose of the CA (*i* = 1, 2, …, 7), R_j_ is the random effect of the experiment (experiment), and e_ij_ is the residual effect. An orthogonal polynomial test was conducted using the SAS PROC IML procedure to determine the linear and quadratic effects of *CA.* Data from gas production kinetics and broken-line regression were analyzed using the SAS PROC NLIN procedure. For analyzing the broken line regressions, TVFA was used as the criterion of the model. Graphical depiction of the broken-line regression results was done using the GPLOT procedure in SAS (Figure 1).

The experiment statistical model for the *in vivo* experiment were analyzed using the MIXED procedure of SAS and the statistical models was as follow equation.


$$Y_{ij}=\mu +T_{i}+R_{j}+e_{ij}.$$

Where Y_ij_ is the observation from the experimental unit, μ is the overall mean, T_i_ is the fixed effect of the CA, R_j_ is the random effect of the animal (*i* = 1, 2…12), e_ij_ is the residual effect. All the data differences between the treatments were compared with Tukey’s test. The significance level was declared at *p* < 0.05, and tendency was declared at 0.05 ≤ *p* < 0.1.

## 3 Results

### 3.1 *In vitro* rumen gas and CH_4_ production

**Figure 2:**
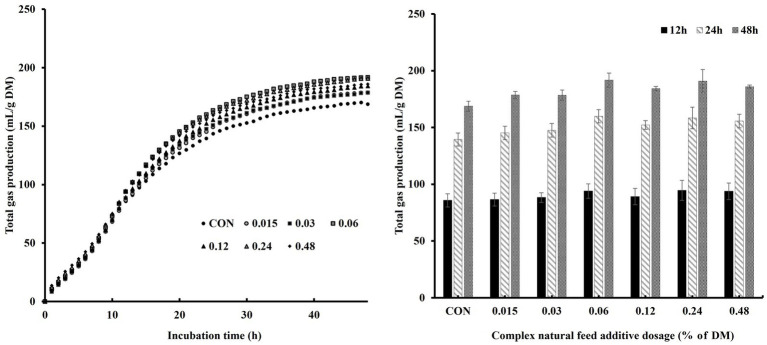
Effects of complex natural feed additives on gas production profiles during *in vitro* rumen fermentation based on incubation time and dosage.

We investigated *in vitro* rumen gas production and CH_4_ production at various levels of CA inclusion concerning incubation time and dosage (Table 2 and Figure 2). There were no significant differences in the fitted parameters of gas production profiles. However, the inclusion of CA tended to increase V_max_ (*p* = 0.05).

**Table 2:** The fitted parameters of gas production profiles.

Parameters^1^	Complex natural feed additive (% of DM)							SEM	*p*-value	Contrast^3^	
	0	0.0015	0.03	0.06	0.12	0.24	0.48			L	Q
V_max_ (mL/g DM)	188.90^b^	200.40^ab^	197.60^ab^	213.90^a^	206.43^ab^	211.20^ab^	205.60^ab^	2.83	0.05	0.11	0.03
K (*h*^−1^)	0.057	0.054	0.057	0.057	0.055	0.057	0.058	<0.01	0.64	0.21	0.94
L (*h*)	0.93	1.24	1.30	1.33	1.22	1.09	0.89	0.06	0.08	0.04	0.19

Means in a row with different superscript letters are significantly different (*p* < 0.05). ^1^V_max_, the asymptotic gas production; K, fractional gas production rate; L, lag time. ^2^SEM, standard error of the mean (*n* = 3). ^3^L, linear; Q, quadratic.

Time-dependent effects of *in vitro* rumen gas production and methane emission were shown in Table 3. The inclusion of CA increased TGP at all incubation times (*p* < 0.05), with the highest observed at the 0.06% level. There were no significant results in methane production, but it tended to decrease when supplemented with CA, especially with the most significant reduction observed at 0.03% of DM inclusion (*p* = 0.07). Methane proportion was found to be lower with CA inclusion at 12 h of incubation (*p* = 0.02).

**Table 3:** Time-dependent effects of complex natural feed additive on *in vitro* rumen gas and methane production.

Parameters^1^		Complex natural feed additive (% of DM)							SEM^2^	*p*-value	Contrast*^3^*	
		0	0.0015	0.03	0.06	0.12	0.24	0.48			L	Q
TGP (mL/g DM)	12 h	85.77^b^	86.46^ab^	88.27^ab^	93.92^ab^	89.10^ab^	94.55^a^	93.76^b^	1.67	0.02	<0.01	0.06
	24 h	139.4^b^	145.24^ab^	147.47^ab^	159.83^a^	152.2^ab^	158.42^ab^	155.62^ab^	2.10	0.03	0.02	0.03
	48 h	168.76^b^	178.68^ab^	178.52^ab^	191.73^a^	184.28^ab^	190.86^a^	185.99^ab^	2.05	<0.01	0.02	<0.01
CH_4_ (mL/g DM)	12 h	20.01	19.55	17.62	19.20	18.91	19.45	19.46	0.40	0.07	0.53	0.53
	24 h	30.41	30.25	33.75	33.86	33.94	33.79	32.47	0.69	0.17	0.40	0.04
	48 h	43.47	45.07	47.40	46.88	46.90	48.77	48.86	0.63	0.22	0.03	0.21
CH_4_ proportion (%)	12 h	23.4^a^	22.71^ab^	19.94^b^	20.62^ab^	21.23^ab^	20.59^ab^	20.77^ab^	0.43	0.02	0.07	0.05
	24 h	21.75	20.77	22.88	21.18	22.30	21.34	20.78	0.34	0.27	0.26	0.44
	48 h	25.71	25.17	26.58	24.49	25.74	25.58	26.27	0.27	0.54	0.44	0.63

Means in a row with different superscript letters are significantly different (*p* < 0.05). ^1^TGP, Total gas production; CH_4_ proportion, methane to total gas production ratio. ^2^SEM, standard error of the mean (*n* = 3). ^3^L, linear; Q, quadratic.

### 3.2 *In vitro* rumen fermentation parameters

After 48 h of incubation, we investigated the rumen fermentation characteristics with various levels of CA added (Table 4). Ruminal pH was not significantly affected by the inclusion of CA in the ruminal fluid. TVFA production tended to increase in the treatment groups (*p* = 0.07), especially the molar proportion of butyrate, which increased in all the treatment groups (*p* < 0.01). Iso-butyrate and iso-valerate also increased in the treatment groups (*p* = 0.01). Correlating with these results, branched-chain fatty acids (BCFA) were also increased in the treatment group (*p* = 0.01).

**Table 4:** Effects of complex natural feed additive on rumen fermentation characteristics (48 h).

Parameters^1^	Complex natural feed additive (% of DM)							SEM^2^	*p*-value	Contrast^3^	
	0	0.0015	0.03	0.06	0.12	0.24	0.48			L	Q
pH	6.74	6.69	6.70	6.67	6.68	6.68	6.70	0.01	0.05	0.59	0.03
IVDMD (%)	79.57	80.18	80.30	82.98	82.01	80.97	80.23	0.38	0.12	0.88	0.06
NH_3_-N (mg/dL)	117.27	112.85	106.49	116.63	110.37	112.24	106.10	1.98	0.34	0.15	0.79
TVFA (mM)	41.61	44.31	44.82	46.40	45.69	46.06	45.01	0.48	0.07	0.20	0.03
Acetate (mM)	24.99	25.64	25.99	26.35	25.92	26.69	26.10	0.19	0.36	0.22	0.11
Propionate (mM)	9.13	9.84	10.35	10.15	10.16	10.14	9.83	0.17	0.34	0.60	0.10
Iso-butyrate (mM)	0.34^b^	0.38^ab^	0.37^ab^	0.39^ab^	0.41^a^	0.39^ab^	0.40^a^	0.01	0.02	0.01	0.03
Butyrate (mM)	5.05^b^	5.49^ab^	5.65^ab^	6.06^a^	5.91^a^	5.96^a^	5.91^a^	0.09	<0.01	0.01	0.01
Iso-valerate (mM)	1.43^b^	1.86^a^	1.67^ab^	1.83^a^	1.85^a^	1.76^ab^	1.73^ab^	0.04	0.01	0.50	0.06
Valerate (mM)	1.02	1.15	1.10	1.16	1.16	1.15	1.10	0.02	0.05	0.74	0.04
BCFA (mM)	1.76^b^	2.18^a^	2.04^ab^	2.22^a^	2.26^a^	2.17^a^	2.13^ab^	0.05	0.01	0.19	0.02
A:P ratio	2.73	2.61	2.61	2.65	2.59	2.65	2.63	0.03	0.77	0.83	0.70

^ab^ Differing superscripts within a row indicate treatment differences (*p* < 0.05).

^1^IVDMD, in vitro dry matter degradability; NH3-N, ammonia nitrogen; BCFA, iso-butyrate + iso valerate; A:P ratio, acetate to propionate ratio. ^2^SEM, standard error of the mean (*n* = 3). ^3^ L, linear; Q, quadratic.

### 3.3 Broken-line regression analysis

A quadratic effect was observed in the CA inclusion group regarding TVFA production (*p* = 0.03). The TVFA was used as the criterion for broken-line regression analysis. Quadratic broken-line regression analysis estimated that the maximum TVFA was produced at 0.0703% DM inclusion (*Y* = 46.2021–684.7 (x - 0.073)^2^ (x > 0.073), *Y* = 46.2021–2.6937x (x < 0.073); *r*^2^ = 0.37; *p* = 0.045) (Figure 1).

### 3.4 Growth performance

The effects of CA supplementation on the growth performance of Korean-native steers are shown in Table 5. The average daily gain (ADG) was decreased in treatment group compared to control group (*p* = 0.01). Additionally, the feed gain ratio tended to decrease in treatment group (*p* = 0.07).

**Table 5:** Growth performances of Korean-native steers supplemented complex natural feed additive.

Parameters^1^	Control	Treatment (CA)^2^	SEM^3^	*p*-value
Initial body weight, kg	638.76	645.04	13.33	0.85
Final body weight, kg	787.27	754.92	16.44	0.35
ADG, kg/d	0.62^a^	0.46^b^	0.03	0.01
Dry matter intake (kg/d)	10.92	10.41	0.03	0.49
Feed:gain	18.25	24.40	1.78	0.07
Carcass month	32.19	32.25	0.20	0.92

^ab^ Differing superscripts within a row indicate treatment differences (*p* < 0.05). ^1^ ADG, Average daily gain. ^2^ Treatment, complex natural feed additive containing (Probiotics, Red ginseng by-product and C. militaris) supplementation at 0.07% DM. ^3^ SEM, Standard error of means (*n* = 23).

### 3.5 Serum metabolites

The effects of CA supplementation on serum metabolites of Korean native steers are shown in Table 6. No significant differences were observed in serum metabolites between control and treatment group (*p* > 0.05).

**Table 6:** Serum metabolites of Korean-native steers supplemented complex natural feed additive.

Parameters^1^	Control	Treatment (CA)^2^	SEM^3^	*p*-value
Total protein (g/dl)	7.53	7.85	0.19	0.41
Albumin (g/dl)	3.92	3.87	0.05	0.58
TCHO (mg/dl)	213.00	214.27	0.09	0.34
Uric Acid (mg/dl)	0.80	0.82	0.02	0.58
Total glyceride (mg/dl)	19.73	20.82	1.94	0.79
NEFA (μEq/L)	191.17	208.83	13.25	0.48
BUN (mg/dl)	16.46	14.39	1.04	0.32
Glucose (mg/dl)	82.72	85.80	4.51	0.73

^1^ TCHO, Total cholesterol; NEFA, Non-esterified fatty acids; BUN, Blood urea nitrogen. ^2^ Treatment, complex natural feed additive containing (Probiotics, Red ginseng by-product and C. militaris) supplementation at 0.07% DM. ^3^SEM, Standard error of means (*n* = 23).

### 3.6 Complete blood cell count (CBC)

The effects of CA supplementation on complete blood cell count (CBC) of Korean native steers are shown in Table 7. No significant differences were observed in CBC between control and treatment group (*p* > 0.05).

**Table 7:** Complete blood cell count of Korean-native steers supplemented complex natural feed additive.

Parameters^1^	Control	Treatment (CA)^2^	SEM^3^	*p*-value
WBCs 10^9^ /L	8.57	8.00	0.56	0.82
RBCs, 10^12^ /L	10.82	10.56	0.33	0.70
MCH, pg	17.16	17.35	0.17	0.60
MCHC, g/dL	35.32	35.04	0.17	0.34
HGB, g/dL	18.64	18.22	0.50	0.75
HCT, %	52.79	52.56	1.85	0.95
PLT, 10^9^ /L	220.07	257.55	15.79	0.07
LYM, %	22.10	28.37	3.59	0.33
MO, %	5.83	6.18	0.37	0.62
GR, %	72.04	65.44	3.89	0.35

^1^ WBC, white blood cell; RBC, red blood cell; HGB, hemoglobin; HCT, hematocrit; MCH, mean corpuscular hemoglobin; PLT, platelet; MCHC, mean corpusucular hemoglobin concentration; LYM, lymphocyte; GR, granulocyte.

^2^ Treatment, complex natural feed additive containing (Probiotics, Red ginseng by-product and C. militaris) supplementation at 0.07% DM. ^3^ SEM, Standard error of means (*n* = 23).

### 3.7 Carcass characteristics

The effects of CA supplementation on the carcass characteristics of Korean native steers are shown in Table 8. Backfat thickness was significantly decreased in the treatment group compared to the control group (*p* = 0.03). Meat color tended to increase in treatment group (*p* = 0.08). There were no significant differences in other parameters related to carcass characteristics (*p* > 0.05) between the control and treatment groups.

**Table 8:** Carcass characteristics of Korean-native steers supplemented complex natural feed additive.

Parameters^1^	Control	Treatment (CA)^2^	SEM^3^	*p*-value
Carcass weight, kg	473.00	451.75	10.54	0.34
Loin muscle area, cm^2^	99.91	95.50	2.54	0.41
Backfat thickness, cm	15.55	11.83	0.93	0.03
Marbling score	5.78	6.25	0.33	0.44
Yield index	60.91	61.17	0.55	0.81
Yield grade	19.35	20.83	1.26	0.50
Quality grade	40.00	40.00	1.66	1.00
Meat color	4.46	4.83	0.12	0.08
Fat color	3.00	3.00	0.00	1.00
Texture	2.08	2.00	0.17	0.81
Maturity	2.54	2.58	0.11	0.86

^1^ Yield index = 68.184-[0.625 × back fat thickness (mm)] + [0.130 × rib eye area (cm2)] -[0.024 × carcass weight (kg)]; Yield grade, Converted into numeric value as grade A (high yield; > 67.5) = 30, B (62.0 < yields, < 67.5) = 20, C (low yield; < 67.5) = 10; Quality grade, 1++ (highest) = 50, 1+ = 40, 1 = 30, 2 = 20, 3 (lowest) = 10, Marbling score = No. 1 ~ No. 9 (1 = devoid, 9 = abundant), Meat color, No. 1 ~ No. 7 (1 = bright cherry red, 7 = dark red), Fat color = No. 1 ~ No. 7 (1 = white, 7 = dark yellow), Maturity = 1 ~ 3 (1 = youthful, 3 = mature). ^2^ Treatment, complex natural feed additive containing (Probiotics, Red ginseng by-product and C. militaris) supplementation at 0.07% DM. ^3^ SEM, Standard error of means (*n* = 23).

## 4 Discussions

### 4.1 *In vitro* experiment

The aim of the *in vitro* study was to assess the effects of CA on the rumen environment. Rumen gas production is an indirect measurement of rumen microbial fermentation and activity. Recently, rumen gas production has been applied in the field of ruminant research to estimate microbial fermentation processes (23) and evaluate the effect of anti-nutritional factors on microbial activity (28, 29). Gas production primarily results from the fermentation of carbohydrates in the rumen, yielding acetate, propionate, and butyrate, and releasing final catabolites, mostly carbon dioxide and methane (23).

In this study, total gas production significantly increased in the treatment group at every time-period. This increase in total gas production was observed alongside a tendency for increased VFA production. According to a previous study by Getachew et al. (30), gas production has positive correlation with butyrate production. In this study, butyrate production was positively correlated with total gas production in treatment group in linear regression analysis (not shown in table), which may be attributed to the increased TGP. Consistent with our findings, another study reported that *C. militaris* increased rumen cellulolytic bacteria, specifically *Ruminococcus flavefaciens* in rumen *in vitro* fermentation (13, 31). Rumen bacteria are the most abundant microbes in rumen and approximately 10^10^–10^11^ cells/ml and over 200 species inhabit in the rumen (32). The plant fiber matrix is complex and composed of polymer of carbohydrate, breakdown of fiber requires the coordination of a hydrolytic enzymes (33). Cellulose-degrading bacteria reside in the rumen, the most effective cellulose degraders in the rumen due to their ability to digest cellulose, facilitated by the presence of numerous genes encoding for fiber degradation (33). A series of fermentation processes by cellulolytic bacteria produce VFA in rumen. The reasons for the increased bacterial population in rumen are not known but, it could guess that polysaccharide from *C. militaris* plays energy sources to rumen microbes by manipulating the composition in rumen. Several studies reported that galactomannan provide nutrients for beneficial microbes in the gastrointestinal microbiota (34) and D-mannitol can also become energy sources for bacteria growth in the rumen (35). Also, Cappellozza et al. (36), reported that the inclusion of *Bacillus-*based probiotics can increase rumen *in vitro* gas production due to the production of various enzymes by *Bacillus* sp. The protease produced from *Bacillus. Subtilis* (*B. subtilis*), called subtilisin, may degrade proteins in the rumen, providing peptides and amino acids for microbial crude protein synthesis (37). Not only does *B. subtilis* play a role, but *Saccharomyces. Cerevisiae* (*S. cerevisiae*) also contributes to an increased fermentation status in the rumen. The mechanisms behind the higher VFA concentration in *S. cerevisiae* are not fully known, but it is associated with anaerobic microflora in the rumen (35). *S. cerevisiae* has been reported to act as an oxygen scavenger in the rumen, creating favorable conditions for the activity of various microorganisms (38). Additionally, exogenous enzymes such as xylanase from *S. cerevisiae* have been reported to have positive effects on ruminal fibrolytic bacteria by providing readily usable carbohydrates (39). Therefore, the inclusion of CA could enhance the status of rumen microorganisms through the synergistic effects of bioactive compounds in CA, promoting the production of diverse enzymes and making the substrate more efficient to break down.

Methane production results from the fermentation of digestible structural carbohydrates in the rumen (40). Ruminal methane is produced by methanogens in a reduction pathway of carbon dioxide (CO_2_) and hydrogen (H_2_) (41). In this study, methane proportion tended to decrease at 12 h of incubation. Kim et al. (31) reported that *C. militaris* had an adverse effect on ciliate protozoan populations in rumen *in vitro* experiments during early incubation. They reported that methanogen archaea were significantly decreased at 12 h incubation with low concentrations of *C. militaris* inclusion (0.10 g/L). Additionally, red ginseng contains triterpene saponin and pharmacological compounds (42). Saponins have a chemical structure that can act like soap, interacting with cell membrane components such as cholesterol and phospholipids (43). Especially in the rumen saponins can form complexes within protozoal cell membrane, leading to cell rupture and lysis (44). Rumen protozoa have a symbiotic relationship with methanogen archaea and are known to provide hydrogen as a substrate for methanogens (45). This interaction may affect microbial diversity and fermentation, thus inhibiting methanogenesis in ruminants. Consistent with the results of Hamid et al. (18), who evaluated the red ginseng byproduct (RGP) as a protein source for the rumen and found that RGP significantly decreased methanogen archaea compared to another conventional ingredient. Thus, the decreased methane production in our study may be attributed to the bioactive compounds of the CA acting synergistically to inhibit the growth of protozoa, ultimately leading to lower the methane formation.

BCFA results from the oxidative deamination and decarboxylation steps of branched-chain amino acids (BCAA) during fermentation (46). Cellulolytic bacteria utilize BCFA for the synthesis of BCAA (47). In this study, branched-chain fatty acids significantly increased with CA inclusion, which was associated with increased protein digestibility. Sun et al. (48) reported that *B. subtilis* natto, used in rumen *in vitro* fermentation at a concentration of 1.0 × 10^11^ CFU/g, significantly increased BCFA production. *B. subtilis* could produce proteolytic enzymes, including subtilisin, which promote protein digestion (37). It can be speculated that *B. subtilis* may accelerate the deamination and increase the utilization of isoacid for the synthesis of BCAA.

### 4.2 *In vivo* experiment

Based on the results from the *in vitro* experiment, 0.07% of CA (on a dry matter basis) was selected for *in vivo* dosage because it resulted in the highest production of VFA, and there were no harmful effects on ruminal fermentation. However, contrary to the results of the *in vitro* experiment, there were no significant results in final body weight and DMI between the control and treatment groups. Consistent with our results, previous studies on the same probiotic species did not show a significant improvement in the DMI of ruminants (49–51), while other studies reported an increased DMI of ruminants (52, 53). Discrepancies in results may be due to the variations in the amounts of probiotics fed, the species of animals, or the age of the animals.

In this experiment, we used red ginseng by-product as a component of the feed additive. RGP is known to contain compounds that can reduce feed intake due to its astringent and irritating taste (54). Also, triterpenoid saponins have been reported to inhibit pancreatic lipase activity (1). Yoshikawa et al. (55) reported that triterpenoid saponins from *Camellia sinensis* inhibited pancreatic lipase activity in mice. Furthermore, the glucuronic acid unit at C3 of the aglycone of triterpenoid saponins has been reported to inhibit α-amylase and α-glucosidase activity (56). Therefore, even though there were no significant differences in DMI between the control and treatment groups, we can infer that, despite the increased VFA production observed in the *in vitro* experiment, saponin may have decreased pancreatic enzyme activity, which could have reduced the bioavailability of nutrients in the treatment group, ultimately leading to a decrease in growth and carcass performances.

Additionally, according to Riddell et al. (57), probiotics show their maximum effects during stressful conditions. Past studies have reported that probiotic supplementation during stressful periods in ruminant, such as weaning, dietary shifts, and the beginning of lactation, can significantly affect the performance of ruminant (58). Therefore, in the current experiment, the relatively well-housed environment may have helped prevent stressful conditions, limiting the potential effects on animal performance. Another speculated reason for the lack of effects on growth performance could be compensatory digestion that occurs in the post-ruminal digestive tract. The hindgut accounts for approximately 2% of the cow’s body weight and has been found to contribute to 8 to 17% of the TVFA absorption proportion from the digestive systems of ruminants (59). The hindgut also accounted for an average of total-tract starch disappearance, averaging 2.9% (range = 0.0 to 6.8%) in steers. Additionally, low digestibility can compensate in the hindgut when ruminal digestibility is low (60). Given these reasons, CA supplementation may not increase the growth performances of Korean-native steers.

Back-fat thickness refers to the external fat thickness of the carcass and is one of the less desirable parts of the carcass, as it is the most sensitive part when calculating the yield grade (KAPE, 2019). As shown, back-fat thickness was significantly decreased in the treatment group. Consistent with these results, Geng et al. (61) reported that feedlot bulls fed with yeast, increased triglycerides and free fatty acid levels in blood. Increased serum triglycerides level could elicit from the lipolysis of pre-deposited fat. Furthermore, Kim et al. (62) demonstrated that *Bacillus* probiotics increased lipid oxidative genes in liver and decreased lipid accumulation in mice subcutaneous tissues. Similarly, a previous study reported that *C. militaris* extract (CE), specifically cordyrrole a treatment against obesity induced by a high-fat diet in mice, decreased body weight gain and food efficiency ratio (63). They observed that the size of adipocytes was decreased by CE treatment, and active constituents of *C. militaris* inhibited adipocyte differentiation. Considering the significant reduction in backfat thickness and the increase in serum non-esterified fatty acid (NEFA) levels in the supplementation group that we could estimate that the results may have been influenced by the reasons mentioned above. Also, regarding carcass characteristics, previous studies have reported that higher carcass weight in cattle is associated with increased back-fat thickness (64). Carcass weight affects meat quality through its effect on fattiness. While there are other factors affecting the quality of meat, it’s crucial to consider genetic factors, which can vary depending on the animal species and the level of nutrients (65). Also, it’s important to note that the lipid metabolism of ruminants differs from that of monogastric animals, thus further research is needed.

The color of meat is the most important factor for consumers when purchasing meat (66). Meat color is affected by several factors, such as genetics, animal age, nutritional status, and slaughter conditions (67). Fresh meat typically has a bright color and is used as an indicator of its freshness. In this experiment, meat color tended to decrease in the treatment group. The heavier carcass weight of cattle, due to the deposition of energy content from feed, promotes slower glycogen degradation until slaughtering. Heavier cattle also exhibit improved meat color (L*, a*, and b*) values, indicating slower glycogen degradation until slaughtering (68). This is associated with higher glycogen deposition, which accelerates the formation of lactic acid in the muscle. During the pre-slaughter season of cattle, increased physical activity and stress can lead to a higher demand for glycogen and altered pH in the meat, resulting in a change in color (darkening of the meat). Low acidity in the meat leads to a darker color in the muscle. Consequently, we can infer that heavier carcasses deposit more energy, leading to increased production of lactic acid in the muscle. This change in lactic acid levels affects the lower pH in the meat, resulting in a difference in meat color.

Blood hematology parameters are not affected by the supplementation of CA, which means that feeding 0.07% of CA (DM basis) had no negative effect on the immunological responses in Korean-native steers. This could be due to the CA effects vary depending on the animal’s condition (fluctuation of blood), and the low sample size may have contributed to these results (69). Collectively, these results suggest that supplementation with 0.07% CA did not improve the performance of Korean-native steers. In general, the compounds of CA exert their maximum effectiveness in stressful environments, while this study was conducted in a normal environment. Therefore, further studies are suggested to investigate the effects of these factors under diverse conditions for ruminants, especially in stressful conditions.

## 5 Conclusion

Taken together, CA showed no harmful effects on rumen fermentation when supplements were up to 0.48%. However, supplementation of a complex of natural feed additives on Korean native steers significantly decreased average daily gain and back-fat thickness. In addition, there were no significant effects on final body weight, serum metabolites, or the health status of Korean native steers. Therefore, to overcome the limitations of this study, we suggest conducting further research under stressful environments and experimenting with various conditions for animals to determine the efficacy of the feed additives and find ways to enhance their effectiveness.

## Data availability statement

The original contributions presented in the study are included in the article/supplementary material, further inquiries can be directed to the corresponding author.

## Ethics statement

The animal study was approved by Institute of Animal Care and Use Committee of Konkuk University (approval no. KU22079). The study was conducted in accordance with the local legislation and institutional requirements.

## Author contributions

M-SJ: Conceptualization, Data curation, Investigation, Methodology, Project administration, Writing –review & editing. Y-HJ: Methodology, Project administration, Writing – original draft. Y-RK: Data curation, Writing – original draft. JG: Writing – review & editing. J-GL: Resources, Writing – original draft. H-GL: Conceptualization, Supervision, Writing – review & editing.

## Glossary

ADF: Acid detergent fiber
ADG: Average daily gain
aNDF: a-amylase treated neutral detergent fiber
BCFA: Branched chain fatty acid
BCAA: Branched chain amino acid
BUN: Blood urea nitrogen
CA: Complex natural feed additive
CBC: Complete blood cell count
*C. militaris*: *Cordyceps militaris*
CMS: Condensed molasses soluble\
DFM: Direct fed microbial
DMI: Dry matter intake
Exp: the exponential function
IBW: Initial body weight
IVDMD: *In-vitro* dry matter digestibility
K: fractional rate of gas production (h^−1^)
L: discrete lag time (h)
NEFA: Non esterified fatty acids
RGP: Red ginseng by-product
TCHO: Total cholesterol
TGP: Total gas production
TVFA: Total volatile fatty acid
V_max_: The asymptotic gas production (h^−1^)
